# Acoustic emission characteristics and energy evolution law of rock damage process of different pore structures under cyclic loading

**DOI:** 10.1038/s41598-024-52140-1

**Published:** 2024-01-22

**Authors:** Haowen Jiang, Jiandong Dang, Gang Chen, Xiaojun Wang, Kexi Li, Zinan Chen, Shirong Cao, Jian Liu

**Affiliations:** 1 School of Emergency Management and Safety Engineering, Jiangxi University of Science and Technology, Ganzhou, 341000 China; 2 Anhui Tongguan (Lujiang) Mining Co., Ltd, He Fei, 231500 China; 3 School of Resources and Environmental Engineering, Jiangxi University of Science and Technology, Ganzhou, 341000 China; 4 Jiangxi Key Laboratory of Mining Engineering, Jiangxi University of Science and Technology, Ganzhou, 341000 China; 5 Masteel Group, Zhangzhuang Mining Co., Ltd., Lu’an, 237484 China

**Keywords:** Engineering, Materials science

## Abstract

The AE and damage characteristics of three types of pore-structured rock under the same working conditions are studied by means of uniaxial cyclic loading and unloading tests. The results suggest that with repeated loading and unloading, AE ringing increases as a “jump”, and the denser the structure, the earlier the “jump” occurs. The AE cumulative energy shows a “step” upward trend, but there is a significant difference in the “step” spacing. By comparing the energy distribution of rocks with different pore structures, it can be seen that the smaller the porosity and the smaller the pore size, the greater the energy input and storage, and the earlier the internal failure. Compared with the other two energy-based damage calculation methods, the damage calculation method defined in this paper is closer to the true internal damage level of the rock loading cycle. The NSE value of the modified damage variable calculation method was significantly improved and it was shown that the dissipated energy before pore compaction is the main energy causing damage, after pore compaction the combined effects of dissipated energy and plastic deformation energy result in rock damage.

## Introduction

At present, the shallow mineral resources are decreasing or even depleting year by year, and the exploitation of metallic and non-metallic mineral resources is in the stage of comprehensive advancement to the deep part^1^. The mining^2,3^ and blasting^4,5^ processes in rock engineering, and the gas storage and production^6^ processes in salt cavern engineering, are all subject to cyclic loads that deform the rock. Rocks are the basic components of engineering rock masses, and different engineering projects have different rock types. The internal microstructure of different rocks varies considerably, resulting in different mechanical properties and damage characteristics when subjected to external forces. For example: On the basis of the process of rock fracture failure, a new hypothesis of the mechanism of rock fracture has been put forward and verified experimentally^7^; When subjected to cyclic loading, the composite samples exhibit a linear increase in peak stress and Young’s modulus, which correlates with an increase in rock robustness and fracture resistance, but the rate of energy build-up decreases as the rock strength increases^8^; Under triaxial cyclic loading, the salt rock undergoes strain hardening during the initial loading phase, resulting in significant irreversible deformation, and the deviator stress has a direct effect on the magnitude of the residual stress^9^; Using the uniaxial compression test, the researchers investigated the different damage characteristics of the rocks and found that increased strength and reduced porosity increased the susceptibility of the rock samples to fatigue damage^10^; Under the water-mechanical coupling effect, the permeability of granite is intimately linked to the microcrack propagation in the rock. Cyclic loading not only causes fatigue damage. It also limits its fatigue behaviour^11^; Under cyclic loading, more plastic strain and energy loss occurs in the initial loading stage of mudstone, and the damage factor reflects the relationship between fatigue damage and loading^12^; Different damage models can be built according to acoustic emission parameters for limestone with different rockburst tendencies^13^; There are significant differences in the AE and fracture modes of marble in different experiments^14^; Coal, sandstone and shale exhibit different fracture patterns as water velocity increases^15^; A priori cyclic damage affects the robustness and deformation characteristics of rocks, as well as their failure modes^16^; Previous repeated loading influences the deformation of the granodiorite volume, the loss of stiffness, the development of damage and the failure mode^17^. The above researchers have carried out extensive research into the mechanical properties and damage characteristics of rocks under the action of external forces. However, differences in internal grain properties, porosity and pore dimensions between rocks of different lithologies have a direct impact on their mechanical properties and damage characteristics. Therefore, due to the diversity of rock internal structure, uniaxial cyclic loading tests were performed on rocks with different pore structure characteristics. The AE characteristics, energy conversion laws and damage evolution in rocks with different pore structures under identical loads have been studied.

Meng^18^ investigated the effects of different height/diameter ratios on the mechanical properties and damage characteristics of rock specimens, and revealed the influence of size effect on energy. Guo^19^ the mechanical properties and damage characteristics of various predamaged (PD) rocks have been investigated under triaxial compression conditions. Zheng^20^ performed cyclic tests on rocks with different joint roughness coefficients and inhomogeneous Barton standard cross-sections, and analysed the internal energy changes of rocks affected by the discontinuous surface deformation characteristics of the samples. Su^21^ studied the AE and damage characteristics of coal and rock under cyclic loading. Carpinteri^22^ investigated the characteristics and correlations of release, dissipation and emission energy using three-point bending and compression experiments. Meng^23^ investigated the effect of temperature and confining pressure on rock energy evolution using triaxial experiments. The evolution of energy dissipation and damage in salt rocks under the action of uniaxial cyclic stress was studied by Zhu^24^. Li^25^ conducted periodic load/unload experiments on fully saturated limestone under varying loading conditions and discovered the correlation between plastic strain, properties and energy dissipation. Li^26^, based on the uniaxial compression test, investigated the AE characteristics of different rocks at different stages. Carpinteri^27^ used AE monitoring techniques to assess the stability of multi-storey structures, analysed the evolution of damage using AE parameter data collected in experiments, and used these parameters to identify and investigate damage in specimens. Wu^28^ carried out a uniaxial compression test on the masonry specimens and investigated the relationship between the acoustic emission parameters and the damage. Jing^29^ has proposed a new noise cancellation method to analyse the acoustic emission signal after noise cancellation, which avoids modal mixing and improves efficiency. Zhang^30^ found that coal rocks of different burial depths exhibited different mechanical properties and damage patterns when subjected to triaxial cyclic loading.

In summary, there has been a great deal of research into the AE characteristics and damage evolution of rocks during loading. However, there are few studies of the AE characteristics and damage evolution of rocks with different pore structure characteristics under cyclic loading. Various engineering endeavours involve different types of rock, with cyclic loading being the primary method of rock excavation in engineering. Therefore, the acoustic emission characteristics and energy evolution of rocks with different pore structures during the loading process have been studied using a uniaxial cyclic loading test. New damage variable calculation expressions were defined to analyse the influence of pore structure on rock damage evolution. This research aimed to identify mechanisms of damage, deterioration and instability in different engineering rocks under identical conditions.

## Preparation of specimens and test methods

### Preparation of the sample

The lithology of this test is represented by limestone, granite and red sandstone and the sample is from the same hole depth from different mines. Ensure that surface and axial irregularities are within ± 0.02 mm and use an acoustic monitor to check uniformity and integrity. Table 1 shows the physical parameters.

**Table 1:** Basic physical parameters of rock.

Lithologic characters	Numbering	Height/mm	Diameter/mm	Density/g cm^−3^	Longitudinal wave velocity/(m/s)	Average velocity/(m/s)
Limestone	A-1	99.70	50.34	2.74	6207.34	6216.70
	A-2	99.84	49.40	2.74	5997.50	
	A-3	99.66	49.40	2.73	6287.36	
	A-4	99.80	50.10	2.71	6322.74	
	A-5	99.60	49.50	2.72	6278.18	
	A-6	100.00	50.00	2.75	6207.07	
Granite	H-1	98.30	50.10	2.61	3549.05	3436.82
	H-2	98.80	50.06	2.60	3547.16	
	H-3	98.70	50.10	2.61	3536.34	
	H-4	100.30	50.36	2.61	3435.29	
	H-5	100.00	50.44	2.60	3425.76	
	H-6	99.90	50.10	2.58	3127.30	
Red sandstone	S-1	99.30	49.72	2.27	2815.29	2891.07
	S-2	98.54	49.82	2.19	2790.58	
	S-3	98.60	49.82	2.27	2844.59	
	S-4	99.10	49.90	2.25	2956.19	
	S-5	99.12	49.90	2.17	2972.22	
	S-6	98.90	49.92	2.23	2967.55	

### Mechanical test method

**Figure 1:**
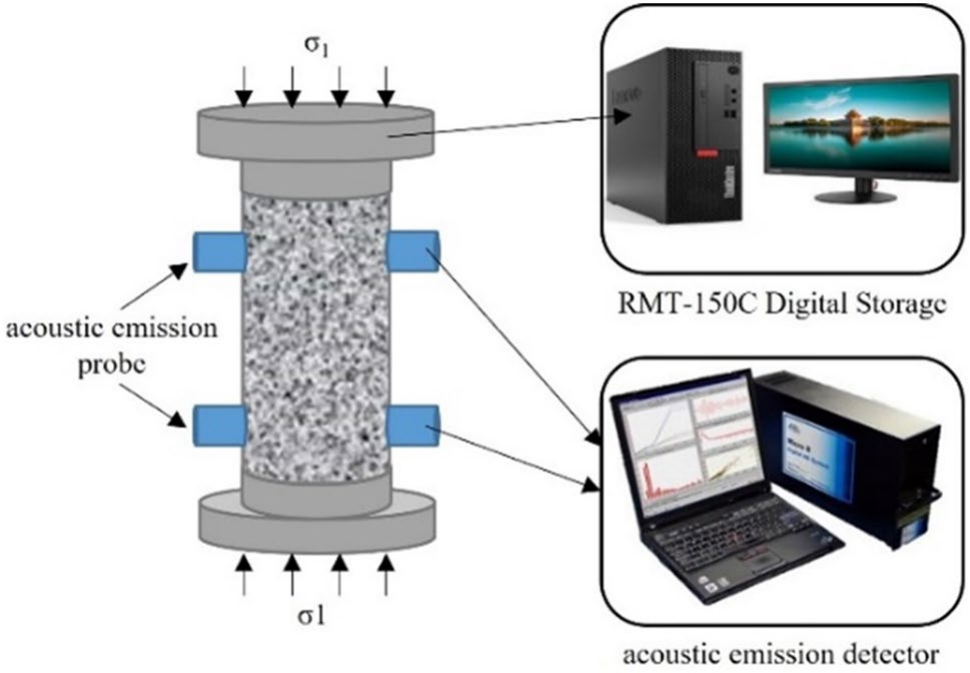
Experiment loading diagram.

**Figure 2:**
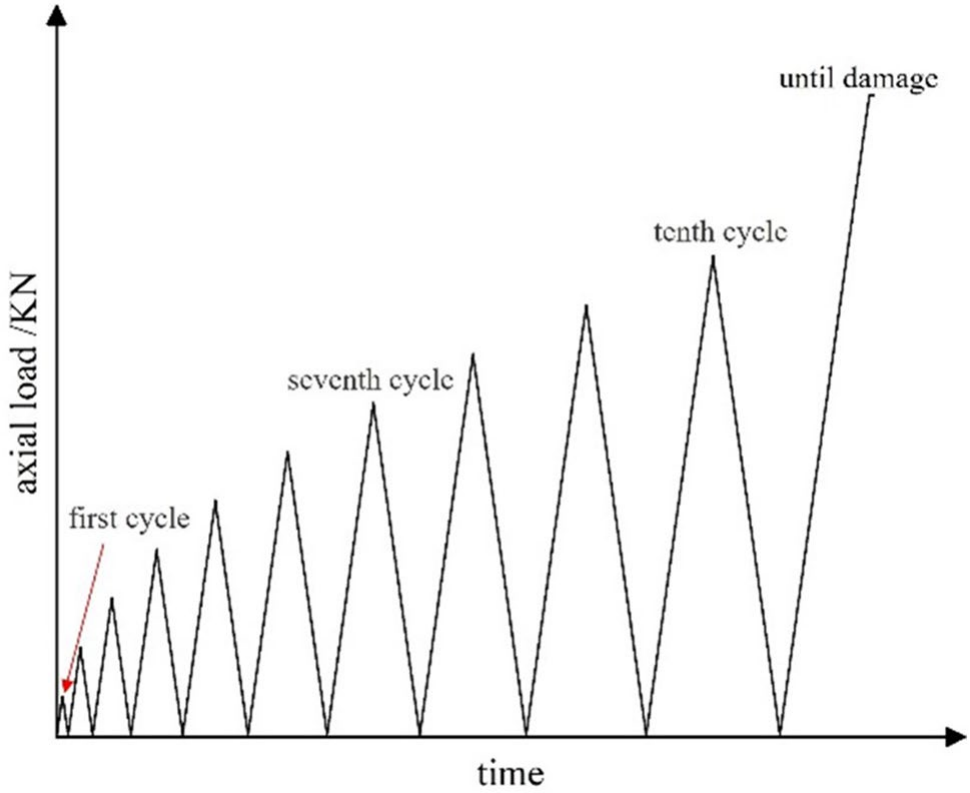
Single-axis cyclic load-and-unload text loading path.

Each lithological sample undergoes a uniaxial compression test to identify the maximum unloading stress points at each stage of each rock sample. The equipment adopts RMT-150C rock mechanics test load control system and PCI-II type AE monitoring system. This load system is capable of applying an axial pressure of 1000 KN and is capable of recording displacement, load, stress and strain values in real-time, as well as drawing stress–strain curves and load-strain curves. Acoustic emission transmission uses a complete set of hardware and software systems to receive, process and display signals. Figure 1 shows a schematic of the test. The test uses stress controlled loading, with a total loading rate of 0.2 KN/s, each rock sample being subjected to 10 cycles of loading and unloading (load increasing in sequence), and if the rock has not broken at the end of the 10th cycle, it will be loaded to rock failure during the next stage of loading. Figure 2 shows the loading path.

## Microstructural and macroscopic properties

### Microstructural features

**Figure 3:**
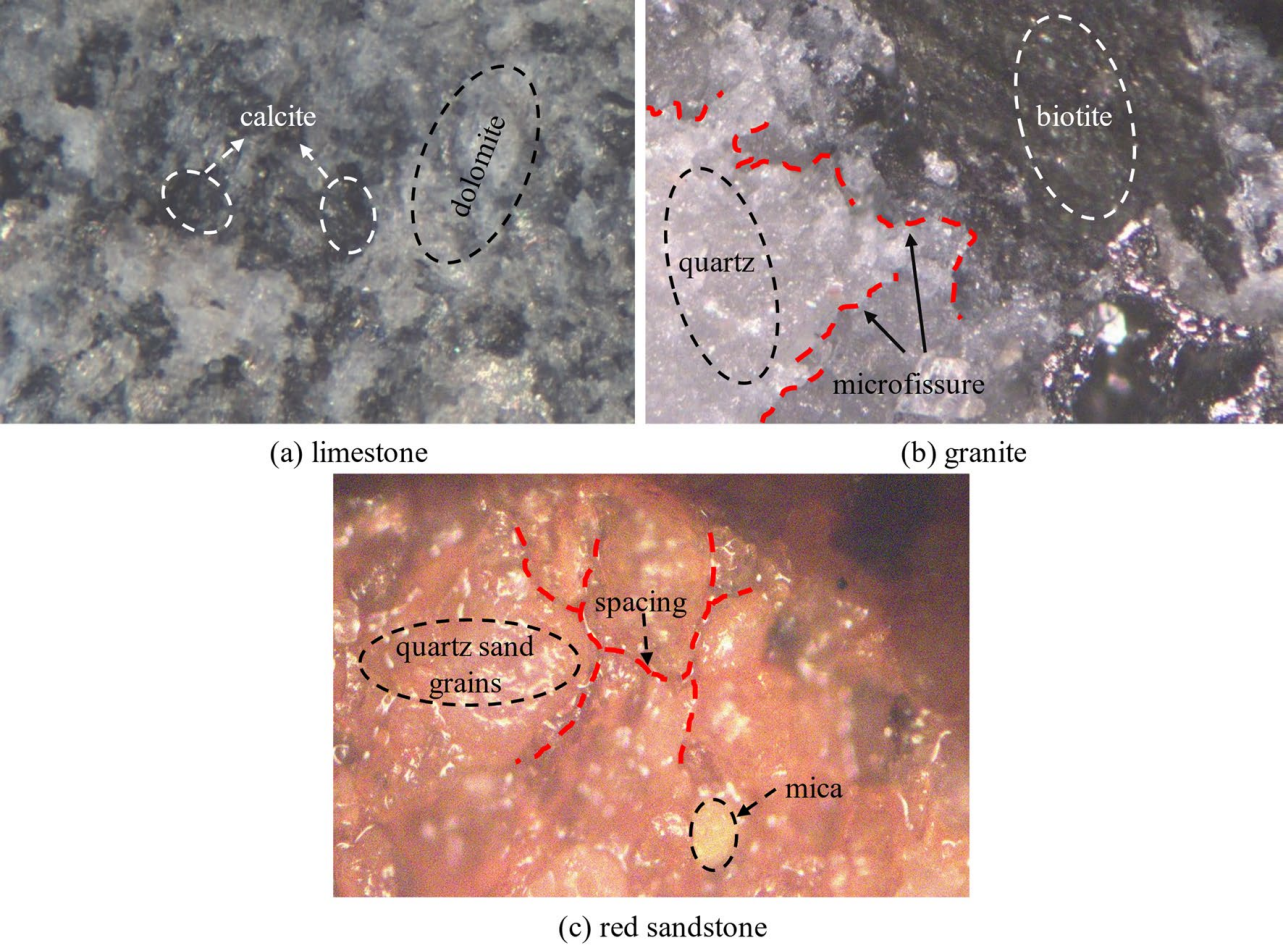
Schematic diagram of metallographic imaging of three rocks.

The macroscopic mechanical properties of a rock are significantly influenced by its pore configuration, grain size, mineral composition and the nature of cementation between its minerals, due to the complex conditions of rock formation and the effects of geological tectonic stresses. By imaging rock samples with a metallographic microscope, it is possible to characterize the physical phase of rock minerals, the morphology of mineral formation, pore structure, grain size, and the number, appearance, size, distribution, and spatial state of certain grain defects. The results of surface imaging using a metallurgical microscope on three rock samples are shown in Fig. 3.

Limestone specimen observation area consists of dolomite and calcite two kinds of minerals, minerals alternate distribution, two kinds of mineral particles are small, closely arranged, no obvious defects and cracking. The observation area of the granite specimen is mainly composed of quartz and black mica, mineral particles are small, relatively concentrated, but in the mineral intersection quartz grains have obvious micro defects and micro cracks. The observation area of the red sandstone sample consists mainly of quartz sand particles and mica, two minerals, the particles have a directional distribution but the quartz sand particles are relatively large with obvious voids between particles. These three rocks were found to differ significantly in mineral composition, pore structure, particle size and arrangement. To further compare the pore structures of the three rocks, the porosity of the samples after vacuum saturation was tested using a PQ-001 Nuclear Magnetic Resonance (NMR) instrument. Table 2 shows the porosities of the three rocks.

**Table 2:** Results of porosity determination of three rock samples.

Limestone	Porosity (%)	Average porosity (%)	Granite	Porosity (%)	Average porosity (%)	Red sandstone	Porosity (%)	Average porosity (%)
A-1	1.256	1.182	H-1	1.030	1.013	S-1	13.571	14.117
A-2	1.201		H-2	1.027		S-2	15.492	
A-3	1.211		H-3	0.998		S-3	13.178	
A-4	1.130		H-4	0.991		S-4	13.287	
A-5	1.150		H-5	1.021		S-5	15.457	
A-6	1.141		H-6	1.012		S-6	13.537	

**Figure 4:**
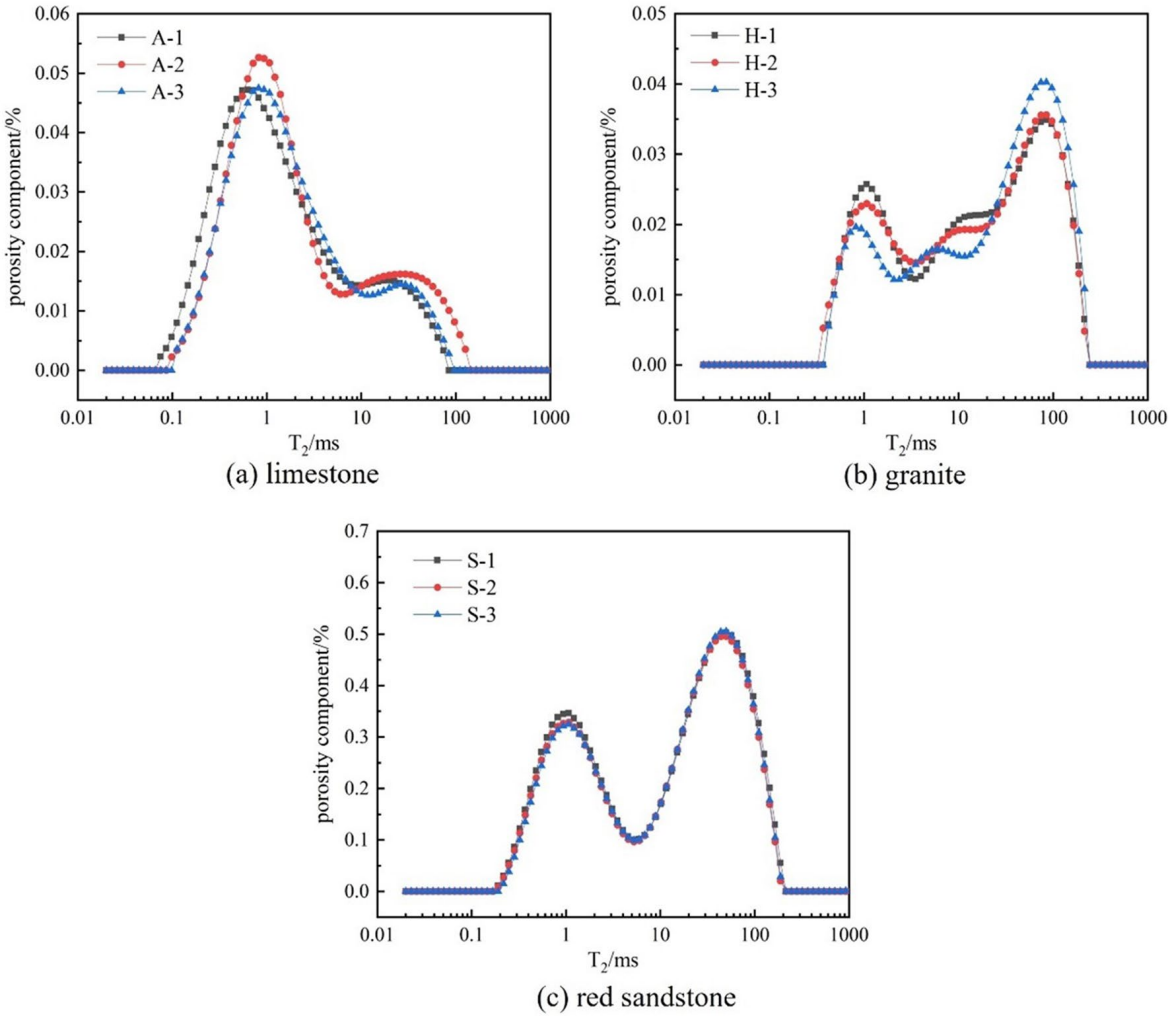
Three types of rock pore component maps.

Table 2 shows that granite samples have lowest mean porosities (1.013%), limestone samples have 1.182% and sandstone samples have highest mean porosities (14.117%). During the porosity test, the T_2_ map of the transverse relaxation time of each rock sample was obtained, and the map could be a reflection of the internal characteristics of the rock^31,32^, T_2_ value size was positively correlated with pore size, with larger T_2_ values indicating larger pores and smaller T_2_ values indicating smaller pores. There is a direct correlation between the T_2_ peak and pore size, with larger peaks indicating increased pore size. The T_2_ atlas of each rock sample is shown in Fig. 4.

From Fig. 4, limestone consists of two spectral peaks, the first peak has a larger area, corresponding to a relaxation time of 0.0934 ms; granite consists of three spectral peaks, the third peak has a larger area compared to the first two peaks, corresponding to a relaxation time of 84.654 ms; red sandstone consists of two spectral peaks, the second peak has a larger area, corresponding to a relaxation time of 49.818 ms. Combined atlas distribution of the three rocks, limestone samples have predominantly small pore size, granite rock samples and red sandstone samples have predominantly large pore size.

Compared with porosity structure of three rock types, limestone specimens have small particles and dense arrangement, and porosity structure is mainly small porosity and small porosity; granitic specimens have small granules and uniform arrangement, but micro flaws and micro cracks exist at intersection of some minerals, and porosity structure is mainly small porosity and big porosity; red sandstone specimens have clear intergranular spaces, and granules have comparatively big size, and porosity structure is mainly big porosity and big porosity. Of these, limestone and granite samples are mostly low porosity, but the pore size of limestone is much smaller than that of granite; both granite and red sandstone samples are mostly high pore size, but the porosity of the latter is much greater than that of the former. In-depth analysis of three types of rock porosity structure, limestone structure is the densest, granite is the second, red sandstone structure is the loosest.

### Macroscopic properties

**Figure 5:**
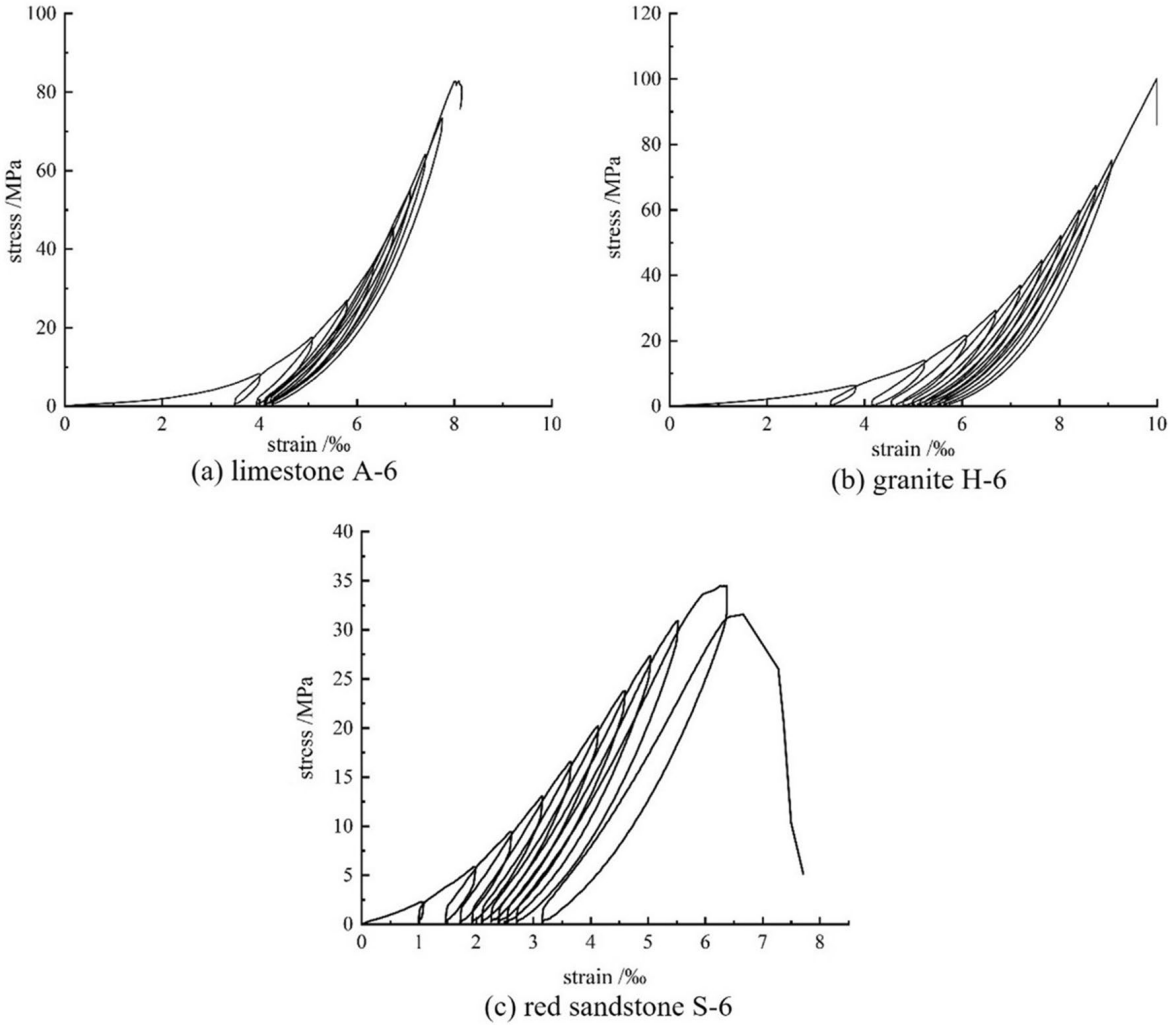
Stress–strain curves of three types of rock subjected to uniaxial cyclic loading and unloading.

The microstructure of geotechnical materials is closely related to their engineering properties. Figure 5 shows the relationship between stress and strain in rocks with different pore structures under cyclic loading conditions.

For rock with high porosity and large pore size, the rock debris generated during loading and unloading fills the pores and creates friction with the pore wall during subsequent loading and unloading, which can prevent damage and indirectly increase the fatigue strength of the rock. Figure 5 shows that the limestone specimen was damaged during the ninth loading cycle, the granite specimen was destroyed during the eleventh loading cycle, the red sandstone specimen was damaged during the reloading process after the tenth loading and unloading cycle, but the failure point did not exceed the stress point of the previous unloading.

Limestone specimens with small porosity and small pore size have a large axial deformation in the pore compaction phase, and after the pore compaction is completed, new cracks are generated earlier and propagation is stable, and the axial displacement of the cyclic load curve is relatively small. Granite specimens with small porosity and large pore size are affected by the pore structure, the pore compaction time is longer, the axial deformation in the pore compaction stage is greater, the crack development is later and more severe, and the axial displacement of the cyclic load curve is greater. For sandstone samples with large porosity and pore size, the external force applied in the initial loading stage is small, resulting in incomplete pore compaction. With cyclic loading and unloading, new cracks are formed in the initial pore compaction, but there are fewer new cracks and less axial deformation. Comprehensive analysis of the cyclic loading patterns in three rock samples shows an escalation in axial strain with the number of cycles in each sample, indicating that the stress point at which unloading occurs in each stage damages the internal structure of the sample.

## Evolution characteristics of rock acoustic emission

**Figure 6:**
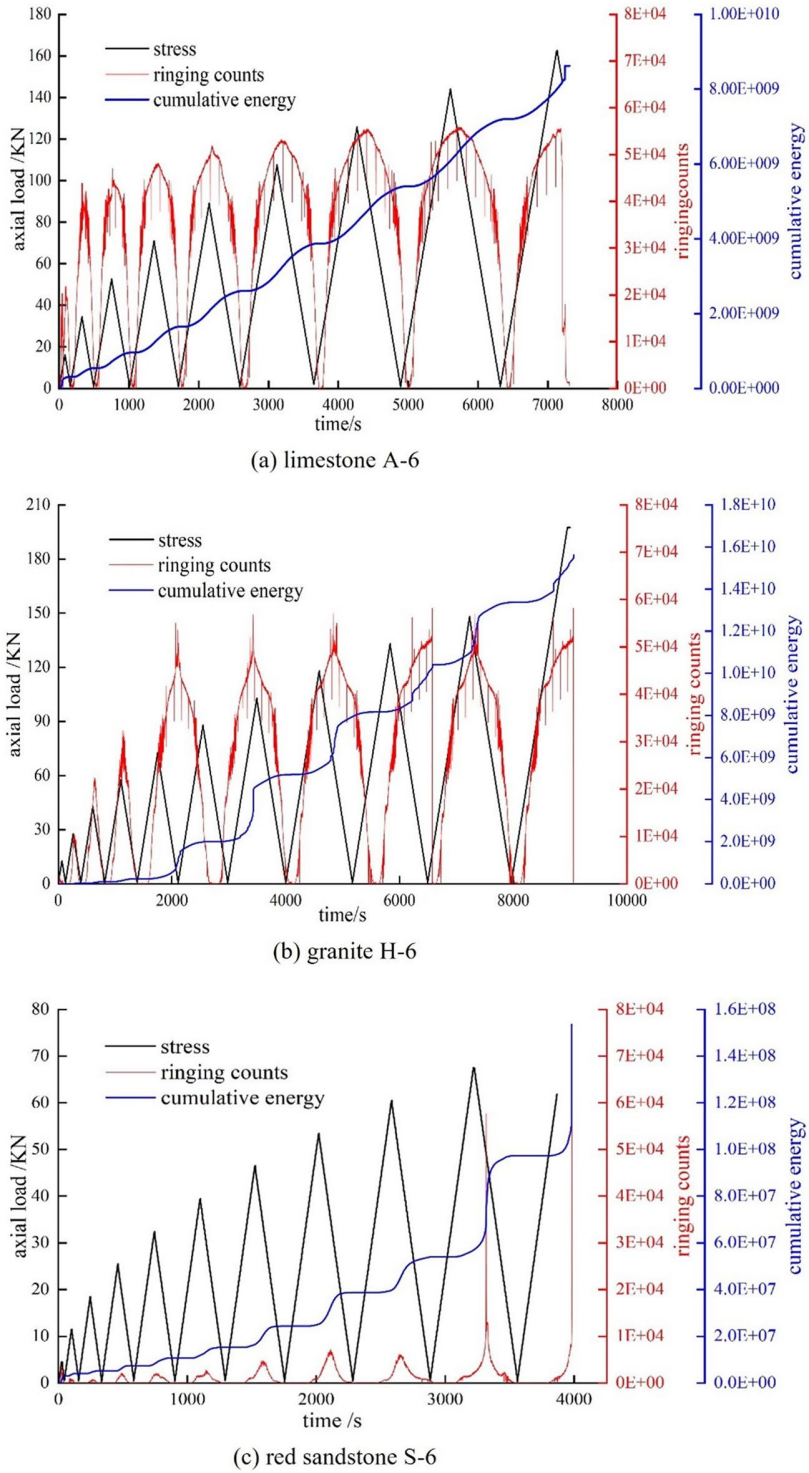
Schematic diagram of axial load-time-acoustic emission ring counting, accumulated energy.

Using AE together with rock compressive deformation data in cyclic load and unload experiments, the study reveals the internal fracture and energy progression laws of rocks under load and unload, thereby indirectly predicting rock instability^33–35^. Figure 6 shows the relationship between stress-time and AE parameters of rocks with different pore structures under cyclic loading.

With axial load and time, the AE ringing number and cumulative energy of rocks with different pore structures vary as follows:

1. Small porosity and small pore size limestone specimen: characterised by a compact structural composition and rapid compression of the pore space after loading. The continuous loading stress leads to the formation of new internal cracks, with a sharp increase in the AE ringing number in the second loading/unloading phase and a similar peak AE ringing number in the subsequent cyclic phase. As the cyclic loading progresses, the cumulative energy of AE increases in a “step” fashion. However, due to the pore structure, the “step” effect is not obvious and the change curve increases approximately linearly. This indicates that damage from cyclic loading is earlier and more stable in the interior of tight rocks.
2. Granite sample with small porosity and large pore size: During the fifth cycle of loading, significant damage occurs in the rock sample resulting in a rapid increase in AE ringing. The crack continues to grow in the following cycle until the rock becomes unstable and damaged, at which point the AE response rate gradually 'moves back' in time with the cyclic loading. When subjected to cyclic loading, the “step” trend of the accumulated energy obviously increases, but the effect of the “step” is more obvious and the accumulated energy shows an increasing trend of “obtuse angle” as a whole. This is an indication that the damage caused by cyclic loading to small porosity but large pore size rock samples is less before pore compaction and the damage caused by pore compaction and sealing is more apparent.
3. Red sandstone specimen with large porosity and large pore size: After compression, the grain deformation space is large and there is no obvious change in the AE ringing count, while the penetration of the crack near the damage point leads to a rapid increase in the emission signal, eventually reaching the peak. The AE ringing count also showed a “backward” trend, and the “backward” trend was more pronounced than in the low porosity rocks. Under the influence of pore structure, the “step” effect of acoustic emission energy accumulation is more obvious. The general trend of acoustic emission energy accumulation is a “power function”. This indicates that cyclic loading–unloading causes continuous damage to rock samples with large porosity and large pore diameter in the initial stage, and the rock samples become unstable and damaged after the pores are gradually penetrated.

In conclusion, the number of AE ringing counts in rocks with different pore characteristics varies with the change in cyclic load curves. For samples with small porosity and pore size, the AE signal is active during the initial loading phase. As porosity and pore size increase, the acoustic emission signal becomes more active before the rock becomes unstable and fails. Under the influence of the pore structure, the AE ringing count gradually “shifts backwards”, and the larger the porosity and the larger the aperture, the more obvious the “backward shift” of the AE ringing count. As the unloading stress gradually decreases, the previously dense porosity and new cracks gradually “rebound”, the accumulated energy increases significantly, the cracks in the heavy load stage become dense again, the accumulated acoustic emission energy tends to be stable and in a dynamic equilibrium state, the accumulated energy shows a trend of increasing “steps”, and the cycle is repeated until the rock becomes unstable and fails. However, there are obvious differences in the “step” effect, and as porosity and pore size increase, the step effect becomes more pronounced, reflecting to some extent the internal failure of the rock.

## Energy distribution law of rocks with different pore characteristics

### Energy calculation method

**Figure 7:**
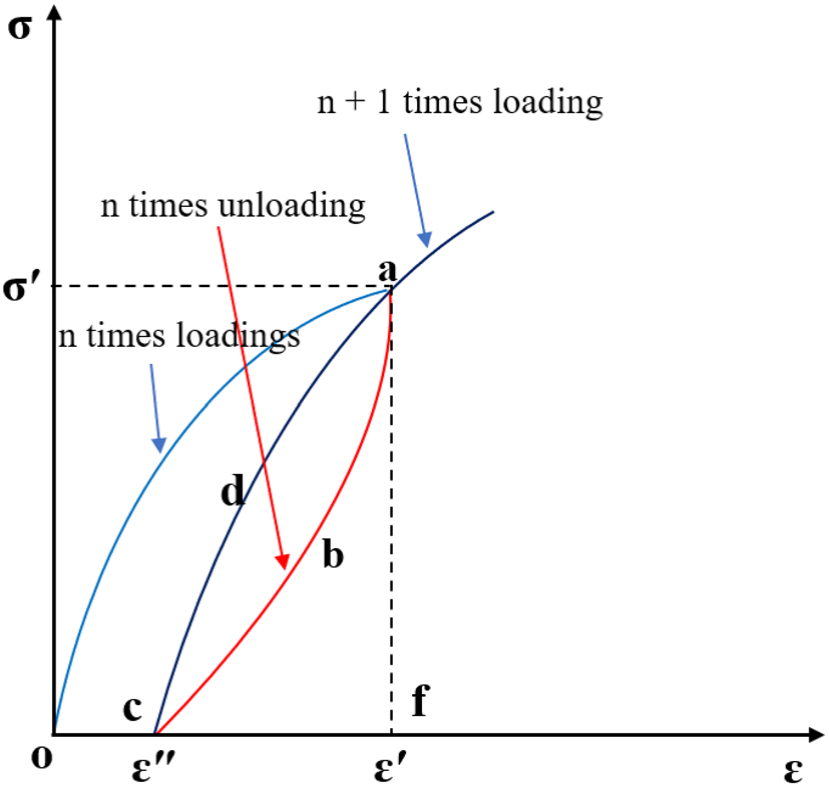
Schematic diagram of the energy calculation in the cyclical load and unload mode.

As the rock is periodically loaded and unloaded, some of the energy from the test machine accumulates in the rock as recoverable strain energy, which is released during unloading, while some is consumed during loading as unrecoverable strain energy^36–38^. Figure 7 shows a typical cyclic load and unload curve and energy calculation diagram:

There is an energy conversion process for loading and unloading rocks. Based on the principle of energy conservation, the energy conversion follows the following equation:

1 $$u={\int }_{0}^{{\varepsilon }{\prime}}\sigma d\varepsilon$$

2 $${u}_{e}={\int }_{{\varepsilon }^{{\prime}{\prime}}}^{{\varepsilon }{\prime}}\sigma d\varepsilon$$

3 $${u}_{d}={\int }_{0}^{{\varepsilon }{\prime}}\sigma d\varepsilon -{\int }_{{\varepsilon }^{{\prime}{\prime}}}^{{\varepsilon }{\prime}}\sigma d\varepsilon$$

The area enclosed by the n + 1 loading curve and the unloading curve of the previous cycle is the dissipated energy, and the plastic deformation energy is the difference between the unrecoverable strain energy and the dissipated energy, i.e. Eqs. 4 and 5:

4 $${u}_{b}={\int }_{{\varepsilon }^{{\prime}{\prime}}}^{{\varepsilon }{\prime}}{\sigma }_{(n+1)}d\varepsilon -{u}_{e}$$

5 $${u}_{c}={u}_{d}-{u}_{b}$$

where *u* is the total energy; *u*_*e*_ is the recoverable strain energy; *u*_*d*_ is the unrecoverable strain energy; *ε'* is the strain value corresponding to the axial load when loaded to σ′; *ε*″ is the strain value corresponding to the axial load gradually relieved from σ′ to 0.

### Trends in energy density of rocks with different pore characteristics

The failure process of rock compression, deformation and instability always involves the input, accumulation, dissipation and release of energy, and the energy and stress, strain and time generated by crack initiation, diffusion and penetration have corresponding functional relationships. Therefore, the internal damage of rocks can be clearly described by studying the energy distribution law under the stress points of each cycle. Table 3 shows the energy distribution of rock specimens with different pore structures during cyclic loading.

**Table 3:** Energy distribution results under cyclic loading and unloading at all levels.

Lithologic characters	Cycle index	Unloading point stress/MPa	Total energy density/(MJ mm^−3^)	Recoverable strain energy density/(MJ mm^−3^)	Unrecoverable strain energy density/(MJ mm^−3^)	Plastic deformation energy density/(MJ mm^−3^)	Dissipated energy density/(MJ mm^−3^)
Limestone	1	8.274	0.055	0.008	0.047	–	–
	2	17.589	0.082	0.037	0.045	0.040	0.005
	3	26.904	0.131	0.082	0.049	0.034	0.015
	4	36.208	0.194	0.140	0.054	0.026	0.028
	5	45.482	0.267	0.206	0.061	0.026	0.035
	6	54.838	0.351	0.280	0.071	0.026	0.045
	7	63.704	0.432	0.352	0.081	0.033	0.047
	8	73.436	0.524	0.425	0.099	0.039	0.060
Granite	1	6.359	0.047	0.005	0.042	——	——
	2	13.988	0.082	0.025	0.057	0.051	0.006
	3	21.657	0.117	0.057	0.060	0.044	0.017
	4	29.276	0.158	0.093	0.065	0.035	0.030
	5	36.936	0.211	0.136	0.074	0.032	0.042
	6	44.565	0.270	0.186	0.085	0.031	0.054
	7	52.194	0.333	0.240	0.092	0.029	0.064
	8	59.823	0.407	0.299	0.107	0.032	0.076
	9	67.462	0.486	0.362	0.124	0.037	0.087
	10	75.132	0.556	0.428	0.128	0.033	0.096
Red sandstone	1	2.307	0.006	0.000	0.005	–	–
	2	5.873	0.019	0.006	0.014	0.013	0.001
	3	9.429	0.034	0.016	0.019	0.014	0.005
	4	13.036	0.055	0.030	0.025	0.014	0.011
	5	16.582	0.080	0.049	0.031	0.014	0.017
	6	20.189	0.108	0.072	0.036	0.013	0.023
	7	23.786	0.150	0.099	0.050	0.025	0.025
	8	27.322	0.190	0.130	0.060	0.027	0.033
	9	30.919	0.240	0.164	0.076	0.033	0.043
	10	34.475	0.356	0.202	0.154	0.083	0.071

**Figure 8:**
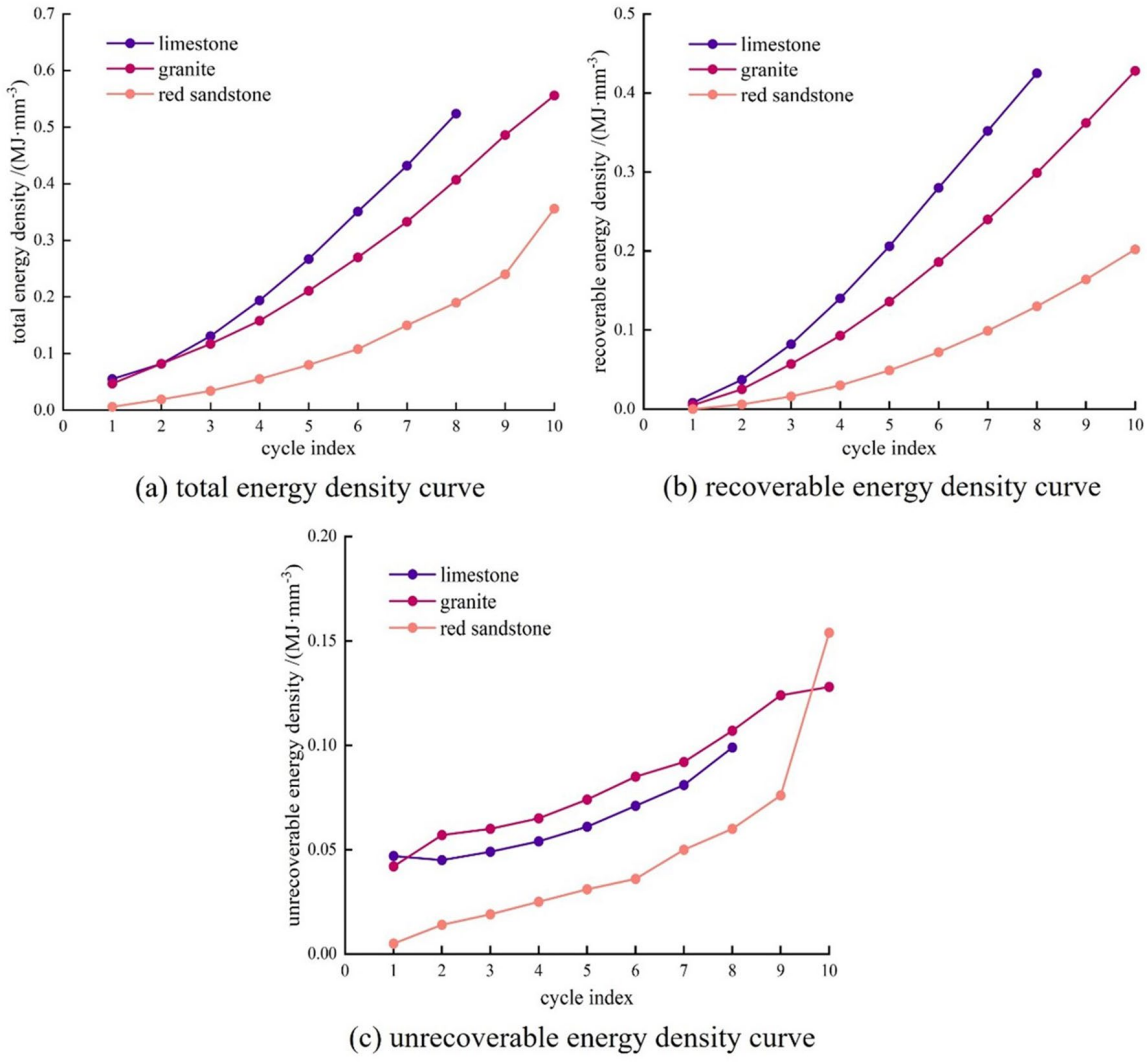
The energy density of the three rocks changes with the number of cycles.

From Table 3 and Fig. 8a,b, the total energy and retrievable strain energy density curves for the trio of rocks showed a non-linear increase pattern in the initial phase and a linear increase in the final phase. According to the linear fit (Table 4), the total energy density curve and the recoverable strain energy density curve for the limestone and granite specimens (slopes of 0.69, 0.61, 0.57 and 0.48) are much larger than those for the red sandstone specimens (slopes of 0.35 and 0.23), indicating that the denser the pore structure of the rock, the more energy is absorbed and stored. At the onset of loading, due to the pore structure, the unrecoverable strain energy density curve of small porosity rocks is high, as shown in Fig. 8c. However, with increasing cyclic loading and number of cycles, a consistent increase in unrecoverable strain energy density is observed across the trio of rocks. This indicates that cyclic loading exacerbates the expansion and development of rock fractures, a process of continuous accumulation of plastic deformation. The limestone rises slowly throughout the phase. The rate of increase in the curve of the granite specimen reached 13.8% during the fifth cycle of loading and unloading, indicating that a relatively large failure occurred during this process. In the final cyclic stage of the red sandstone sample, the unrecoverable strain energy density curve rises abruptly, indicating that fracture propagation and significant failure near the peak have led to rock instability and failure.

**Table 4:** Total energy density-recoverable energy density curve fitting parameters of each rock.

Lithologic characters	Porosity	Pore size	Total energy density Fitting curve	Slope	Correlation coefficient	Recoverable energy density fitting curve	Slope	Correlation coefficient
Limestone	Small	Small	y = 0.69x − 0.055	0.69	0.98	y = 0.61x − 0.085	0.61	0.99
Granite	Small	Larger	y = 0.57x − 0.048	0.57	0.98	y = 0.48x − 0.083	0.48	0.97
Red sandstone	Large	Larger	y = 0.35x − 0.069	0.35	0.90	y = 0.23x − 0.047	0.23	0.95

Calculation of the changes in the proportion of recoverable and unrecoverable strain energy in each rock sample from compressional deformation to instability failure shows that the tighter the structure, the energy stored in the rocks is higher.

### Trend in dissipated energy and plastic deformation energy

**Figure 9:**
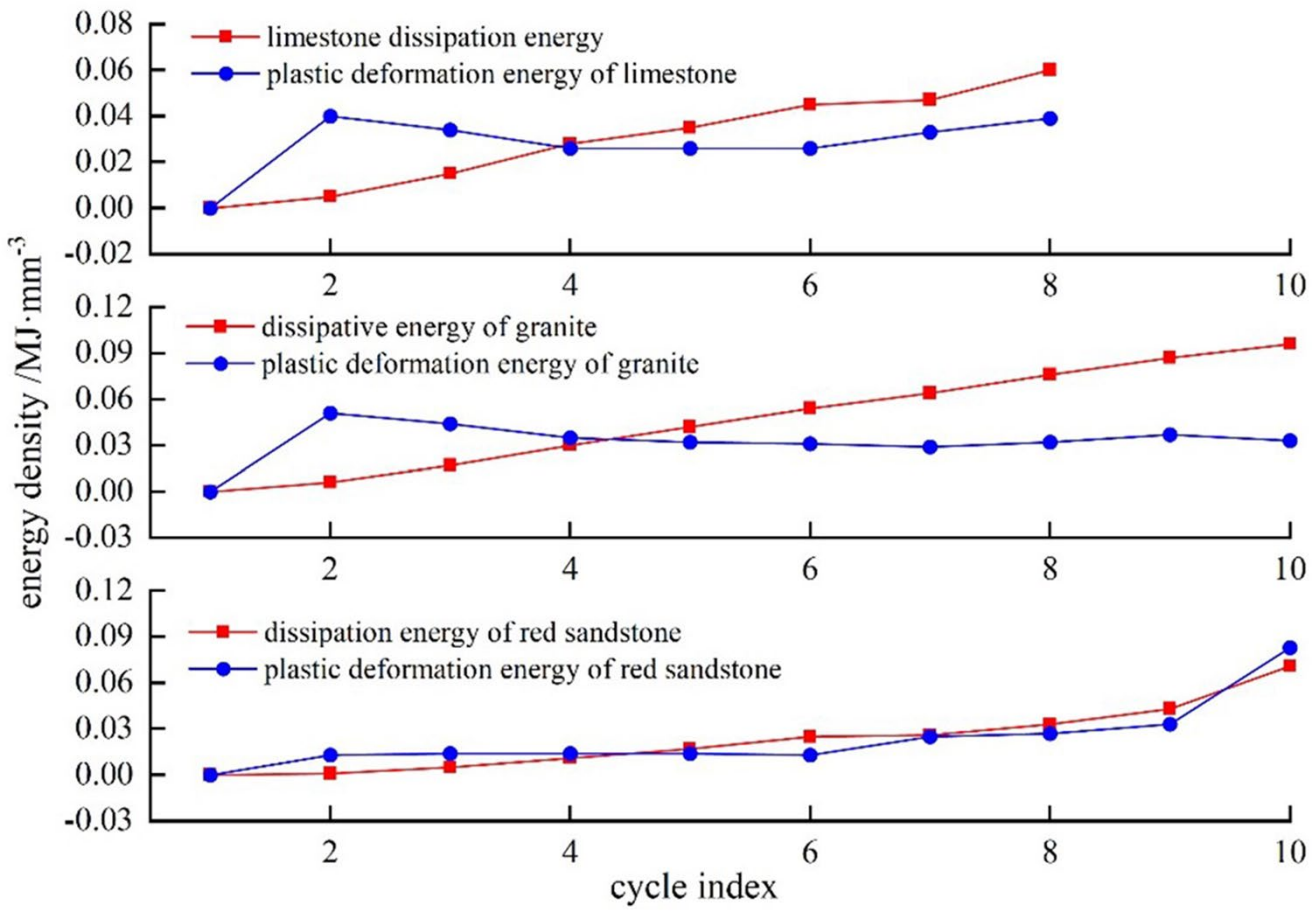
Three types of rock dissipative energy, plastic strain energy-cycle number curve.

Figure 9 shows that with more cycles there's an increase—a decrease—in plastic deformation energy within the trio of rocks, and a steady increase in dissipated energy. The pores are gradually compressed and closed under cyclic loading, causing internal damage to the rock, and the energy dissipated gradually exceeds the plastic strain energy, which becomes the main form of irreversible strain energy. Near the peak intensity, the rock stiffness, internal damage and energy dissipation increase significantly, and the plastic deformation energy increases near the peak strength due to slip dislocation between the internal structural planes of the rock sample.

## Damage evolution characteristics of rock with different pore characteristics

Damage refers to a mechanical behaviour in which cracks and defects gradually develop in the material under load, weakening the material’s cohesion and causing the unit volume to fail. It reflects the deterioration of the macroscopic mechanical behaviour of the material. Scientists study different types of damage variables, including Young’s modulus, energy density and plastic strain, to analyse the characteristics of damage evolution during rock loading. Because rock is affected by external factors and has a lot of microflaws, the damage variables defined in terms of Young's modulus and plastic strain often have diminished characteristics or "negative damage" before the peak intensity^39,40^.

**Figure 10:**
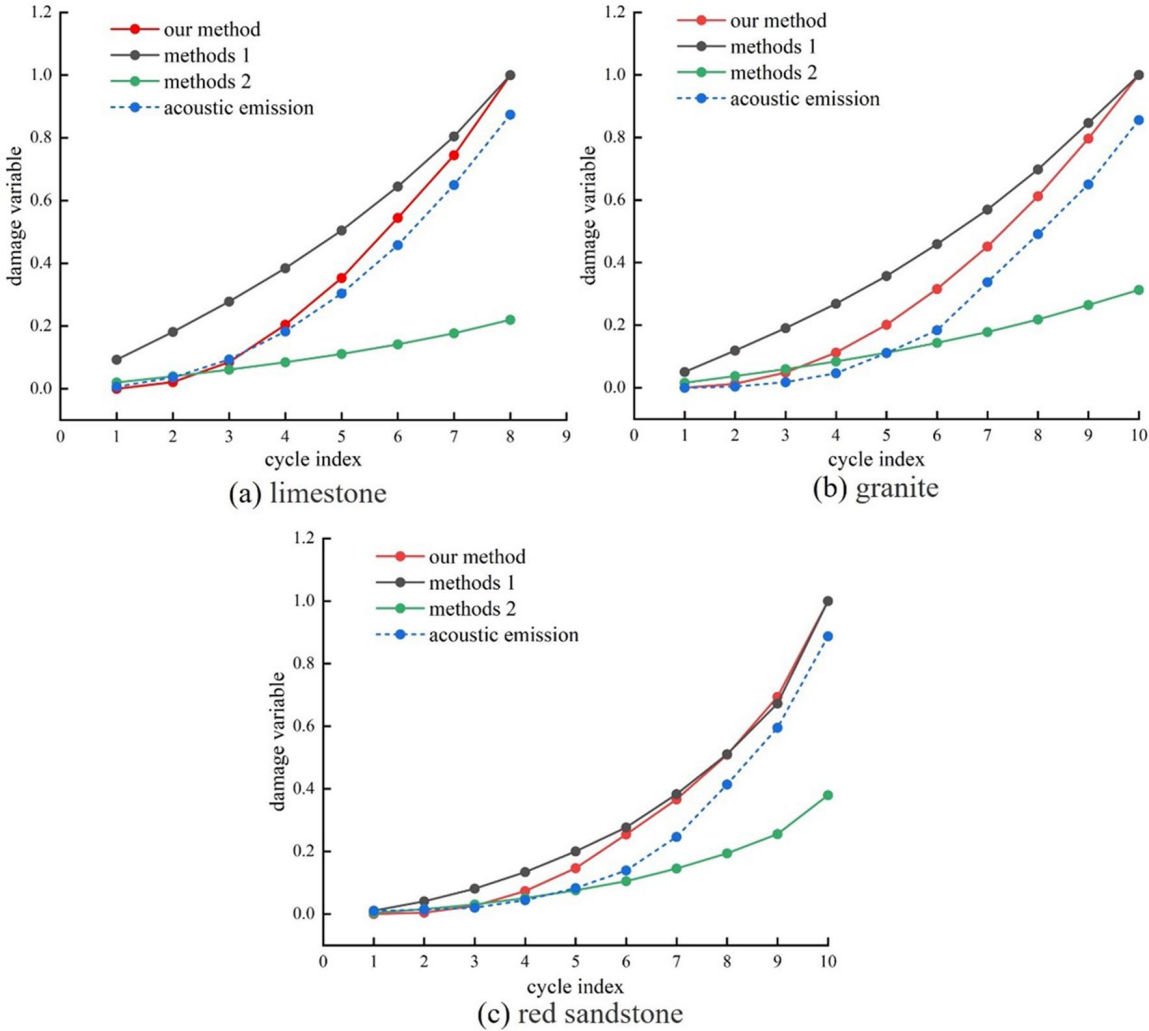
Comparison of damage variables.

From the energy density of the rock, the degree of damage to the rock is determined by the hysteresis characteristics of the stress–strain curve under cyclic loading^41–43^. Based on the study of the energy distribution principle of rocks with different pore structures under cyclic loading in “Energy distribution law of rocks with different pore characteristics” Section, the unrecoverable strain energy is refined and a new type of rock damage variable calculation expression is established. Figure 10 shows a comparative analysis of the results of the damage variables for three different rock cycles compared to the AE theory results:

Method 1^21^:

6 $${D}_{i}=\frac{\sum_{i=1}^{N}{U}_{i}^{d}}{{U}^{d}}$$

Method 2^44^:

7 $${D}_{i}=\frac{\sum_{i=1}^{N}{U}_{i}^{d}}{U}$$

where *U*^*d*^ and *U* are the total unrecoverable strain energy density and the total energy density, respectively, and *N* is the cumulative number of cycles.

The definition of damage in this paper:

8 $${D}_{i}=\frac{\sum_{i=1}^{N}{U}_{i}^{b}}{{U}^{b}}$$

where ${U}_{i}^{b}$ is the density of dissipated energy generated in the ith cycle, ${U}^{b}$ is the total dissipative energy density under cyclic loading, and *N* is the cumulative number of cycles.

Acoustic emission damage^45^:

9 $${D}_{i}=\frac{{N}_{i}}{{N}_{m}}$$

where *N*_*i*_ is the AE cumulative ringing count at the end of the i-th cycle, and *N*_*m*_ is the AE cumulative ringing count at the end of the cycle.

Given the complexity of the rock failure phase in the loading process, it is assumed that the preceding rock failure phase is a complete energy evolution.

Figure 10 illustrates a positive correlation between the damage factors identified in this research and the increase in cycles and loads, suggesting an escalation in the damage progression of the three rock samples as the number of cycles increased. When the damage variables derived from the energy theory are compared with the acoustic emission damage data from three rock samples, the method used to calculate the damage variables in this study is more consistent with the acoustic emission damage results. However, the error between the two damage change curves gradually increases with cycle count and cyclic load. Therefore, according to the analysis in paragraph 5.3 and considering the characteristics of energy change before and after pore compaction, the calculation method of damage variables defined in Eq. (8) in this paper has been revised, and the updated formula is as follows:

10 $${D}_{i}=\frac{\sum_{i=1}^{N}{U}_{i}^{b}+\sum_{i=min}^{N}{U}_{i}^{c}}{{U}^{b}+{U}^{c}} \left\{\begin{array}{c}limestone,min\ge 4\\ granite,min\ge 7\\ red sandstone,min\ge 6\end{array}\right.$$

where ${U}_{i}^{c}$ is the plastic deformation energy density generated at the i-th cycle, and it should be noted that the plastic deformation energy density i-cycle starting point is the cycle corresponding to the lowest plastic deformation energy density of the rock sample; ${U}^{c}$ are the total plastic deformation energy density under cyclic loading.

The Nash Efficiency Coefficient NSE^46^ reflects the degree of agreement between calculated and measured values of the damage variable over time. The better the agreement between the two curves over time, the closer the NSE is to 1.

11 $$NSE=1-\frac{\sum_{i=1}^{N}{\left({Z}_{i}-{D}_{i}\right)}^{2}}{\sum_{i=1}^{N}{\left({Z}_{i}-M\right)}^{2}}$$

where ${Z}_{i}$ is the measured value at the i-th cycle, ${D}_{i}$ is the calculated value at the i-th cycle, *M* is the average of the measured value, and *N* is the number of cycles.

**Figure 11:**
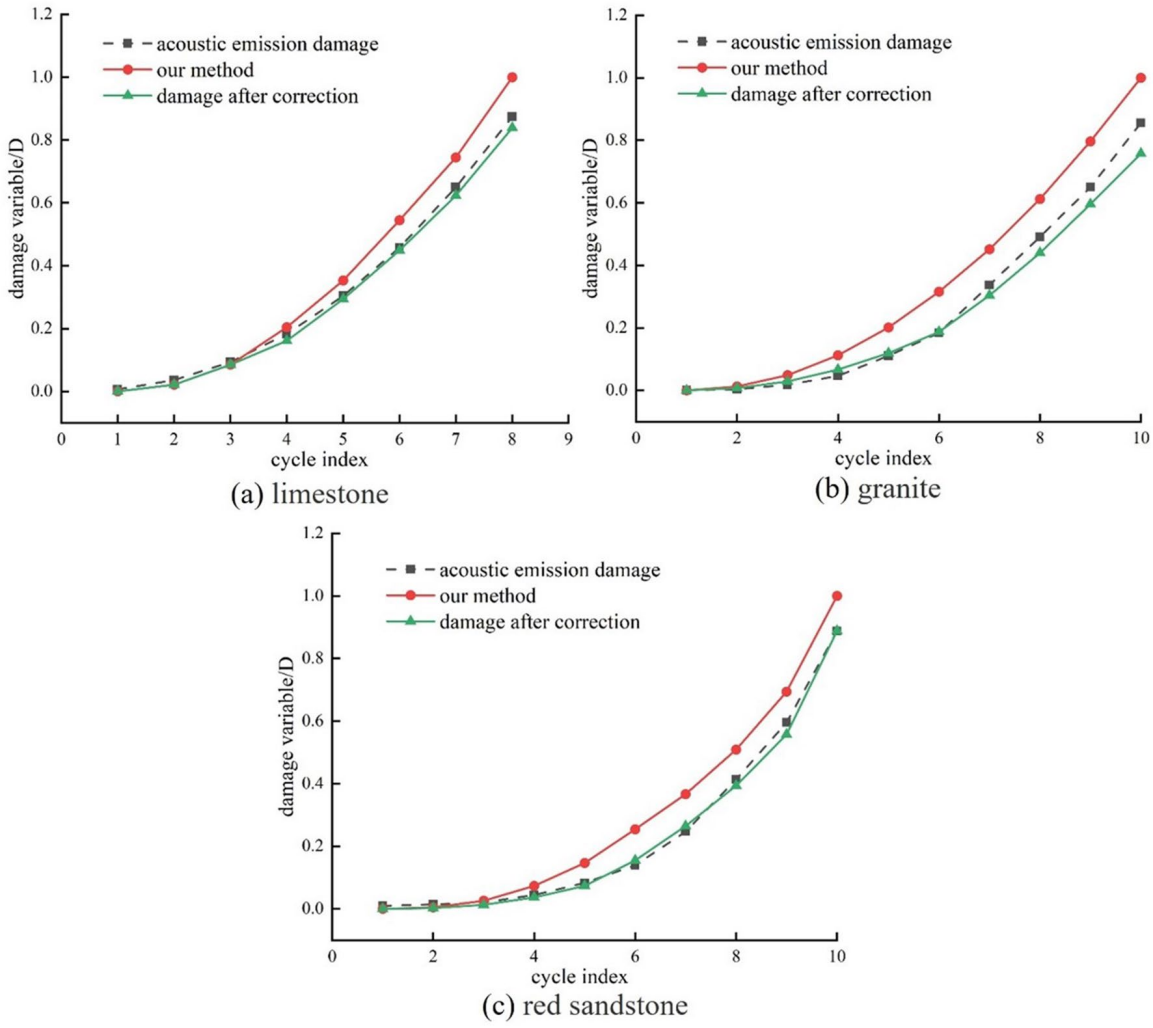
Corrected damage variables.

Figure 11 shows the variation of the damage quantity calculated from Eq. 10 with the number of cycles.

Figure 11 shows that the modified damage variables more accurately reflect the true internal damage of the rock, from load deformation to instability. The goodness of fit over time between calculated and measured damage variables before and after correction was compared using NSE (Eq. 11) as shown in Table 5.

**Table 5:** Results of NSE calculations for rock.

NSE	Lithologic characters		
	Limestone	Granite	Red sandstone
NSE before correction	0.9479	0.8787	0.9199
modified NSE	0.9915	0.9795	0.9817

Table 5 shows that the corrected NSE value has increased significantly. Analysis of the changed NSE values for the trio of rocks shows that a reduction in both porosity and pore size over time improves the correlation between estimated and actual damage values, bringing the NSE value closer to 1. The modified damage variable calculation method shows that the dissipated energy before pore compaction is the main energy causing damage. Post-compaction of pores, the combined effects of dissipation energy and plastic deformation energy result in rock damage.

## Conclusion

This research investigates the AE characteristics, energy distribution and damage evolution in rocks with different pore structure characteristics under uniaxial compression through cyclic loading experiments. The following conclusions have been drawn:

1. From the AE properties of rocks with different pore characteristics: AE ringing counts are found to increase in a “jump” pattern as the pore structure changes, and the denser the structure, the earlier the “jump” occurs and gradually “moves back” as porosity and pore size increase. The accumulated energy shows an upward trend of “steps”, and the “step” effect becomes more significant with increasing rock porosity and pore size, and the distance between “steps” reflects to some extent the internal damage to the rock.
2. The pore structure is shown to have an important influence on the energy conversion of the rock, in combination with the stress–strain curve and the energy distribution law. The dissipation energy exceeding the plastic deformation energy is the point of deterioration of the rock. The lower the porosity and the smaller the pore size, the earlier the rock will fail.
3. The damage variable calculation method based on the dissipative energy definition is close to the actual damage level during rock loading. The modified NSE values show a significant increase in the correlation between estimated and actual losses over time. The smaller the porosity and the smaller the pore size, the closer the NSE value is to 1. The modified damage variable calculation method shows that the dissipated energy before pore compaction is the main energy causing damage, after pore compaction, the combined effects of dissipated energy and plastic deformation energy result in rock damage.

## Data Availability

The datasets used and/or analysed during the current study available from the corresponding author on request.

## References

[1] Wu ZH, Song ZY, Tan J, Zhang YZ, Qi ZJ (2020). The evolution law of rock energy under different graded cyclic loading and unloading modes. Chin. J. Min. Saf. Eng..

[2] Meng QB, Liu JF, Ren L, Pu H, Chen YL (2021). Experimental study on rock strength and deformation characteristics under triaxial cyclic loading and unloading conditions. Rock Mech. Rock Eng..

[3] Yang TJ, Wang PY, Wang SH, Liu H, Zhang Z (2022). Experimental study on shear-seepage coupling characteristics of single fractured rock mass under cyclic loading and unloading. Rock Mech. Rock Eng..

[4] Wang Y, Tang PF, Han JQ, Li P (2023). Influence of dynamic disturbed frequency on rock failure characteristics under triaxial cyclic and multistage unloading confining pressure loads. Fatigue Fract. Eng. Mater. Struct..

[5] Yi L, Feng D (2021). A review of experimental and theoretical research on the deformation and failure behavior of rocks subjected to cyclic loading. J. Rock Mech. Geotech. Eng..

[6] Lin H, Liu J, Yang J, Ran L, Ding G, Wu Z, Lyu C, Bian Y (2022). Analysis of damage characteristics and energy evolution of salt rock under triaxial cyclic loading and unloading. J. Energy Storage.

[7] Bieniawski ZT (1967). Mechanism of brittle bock fracture. Part 1: Theory of the fracture process. Int. J. Rock Mech. Min. Sci. Geomech. Abstr..

[8] Guo WY, Zhang DX, Zhao T, Li YR, Zhao Y, Wang CW, Wu WB (2022). Influence of rock strength on the mechanical characteristics and energy evolution law of gypsum-rock combination specimen under cyclic loading-unloading condition. Int. J. Geomech..

[9] Chen J, Du C, Jiang DY, Fan JY, Yi He (2016). The mechanical properties of rock salt under cyclic loading-unloading experiments. Geomech. Eng. A.

[10] Jeong G-Y, Jang H-S, Jang B-A (2021). Damage characteristics of rocks by uniaxial compression and cyclic loading-unloading test. J. Eng. Geol..

[11] Ning ZX, Xue YG, Li ZQ, Su MX, Kong FM, Bai CH (2022). Damage characteristics of granite under hydraulic and cyclic loading-unloading coupling condition. Rock Mech. Rock Eng..

[12] Xu Y, Li C, Zheng Q, Ni X, Wang Q (2019). Analysis of energy evolution and damage characteristics of mudstone under cyclic loading and unloading. Chin. J. Rock Mech. Eng..

[13] Wang XJ, Zhang H, Chen QL, Zeng Q, Liu J (2022). Acoustic emission characteristics and damage model of limestone loading with different rockburst tendencies. Chin. J. Rock Mech. Eng..

[14] Zha E, Zhang R, Zhang ZT, Ai T, Ren L, Zhang ZP, Liu Y, Lou CD (2020). Acoustic emission characteristics and damage evolution of rock under different loading modes. Energies.

[15] Xiao SQ, Ren QY, Cheng YG, Zhao HY, Cao SR, Zhang L, Chen B, Meng X (2021). Damage and fracture characteristics of rocks with different structures under high-velocity water jet impact. Eng. Fract. Mech..

[16] Xiao F, Jiang DY, Wu F, Zou QL, Chen J, Chen B, Sun ZG (2020). Effects of prior cyclic loading damage on failure characteristics of sandstone under true-triaxial unloading conditions. Int. J. Rock Mech. Min. Sci..

[17] Wang Y, Han JQ, Li P, Cai M (2023). Effect of prior cyclic damage on rock failure exposed to triaxial multistage unloading confining pressure and cyclic loads. Fatigue Fract. Eng. Mater. Struct..

[18] Meng QB, Liu JF, Huang BX (2023). Experimental analysis of the height–diameter ratio effect of rock energy under uniaxial cyclic loading–unloading conditions. Bull. Eng. Geol. Env..

[19] Guo JS, Shang WZ, Yuan J, Liu ZG (2022). Mechanical behaviors and damage evolution characteristics of predamaged rock under triaxial compression experiment. Adv. Mater. Sci. Eng..

[20] Zheng W, Gu L, Wang Z, Ma J, Li H, Zhou H (2022). Experimental study of energy evolution at a discontinuity in rock under cyclic loading and unloading. Materials.

[21] Su ZL, Jing SG, Xie WB, Tang QT (2022). Research on coal acoustic emission characteristics and damage evolution during cyclic loading. Front. Earth Sci..

[22] Carpinteri A, Lacidogna G, Corrado M, Di Battista E (2016). Cracking and crackling in concrete-like materials: A dynamic energy balance. Eng. Fract. Mech..

[23] Meng QB, Liu JF, Huang BX (2022). Effects of confining pressure and temperature on the energy evolution of rocks under triaxial cyclic loading and unloading conditions. Rock Mech. Rock Eng..

[24] Zhu AQ, Liu JF, Wu ZD, Wang L, Liu HJ, Xiao FK, Deng CF (2021). Energy dissipation and damage evolution characteristics of salt rock under uniaxial cyclic loading and unloading tension. Adv. Civ. Eng..

[25] Li J, Hong L, Zhou K, Xia C, Zhu L (2020). Influence of loading rate on the energy evolution characteristics of rocks under cyclic loading and unloading. Energies.

[26] Li S, Zhou M, Gao Z, Chen D, Zhang J, Hu J (2019). Experimental study on acoustic emission characteristics before the peak strength of rocks under incrementally cyclic loading-unloading methods. Chin. J. Rock Mech. Eng..

[27] Carpinteri A, Lacidogna G, Niccolini G (2011). Damage analysis of reinforced concrete buildings by the acoustic emission technique. Struct. Control Health Monit..

[28] Wu YQ, Li SL, Wang DW, Zhao GH (2019). Damage monitoring of masonry structure under in-situ uniaxial compression test using acoustic emission parameters. Constr. Build. Mater..

[29] Jing G, Zhao Y, Gao Y, Marin MP, Lacidogna G (2023). Noise reduction based on improved variational mode decomposition for acoustic emission signal of coal failure. Appl. Sci..

[30] Zhang AL, Xie HP, Zhang R, Gao M, Xie J, Jia ZQ, Ren L, Zhang ZT (2023). Mechanical properties and energy characteristics of coal at different depths under cyclic triaxial loading and unloading. Int. J. Rock Mech. Min. Sci..

[31] Gan Q, Xu J, Peng S (2022). Effect of molecular carbon structures on the evolution of the pores and strength of lignite briquette coal with different heating rates. Fuel.

[32] Ni GH, Dong K, Li S, Sun Q (2019). Gas desorption characteristics effected by the pulsating hydraulic fracturing in coal (Article). Fuel.

[33] Yu C, Yuan HC, Abi E, Han YF, Li HT, Pu YJ (2023). Deformation and acoustic emission characteristics of hard rock under different unloading rates. Alex. Eng. J..

[34] Vinciguerra SC, Greco A, Pluchino A, Rapisarda A, Tsallis C (2023). Acoustic emissions in rock deformation and failure: New insights from Q-statistical analysis. Entropy.

[35] Liu JF, Wang CP, Wang L, Ran LN, Deng CF (2023). Tensile failure and acoustic emission characteristics of rock salt under different tensile testing conditions. J. Cent. South Univ..

[36] Zhang Z, Gao F (2015). Experimental investigation on the energy evolution of dry and water-saturated red sandstones. Int. J. Min. Sci. Technol..

[37] Meng QB, Zhang MW, Han LJ, Pu H, Nie TY (2016). Effects of acoustic emission and energy evolution of rock specimens under the uniaxial cyclic loading and unloading compression. Rock Mech. Rock Eng..

[38] Miao SJ, Xia DH, Yang PJ, Liang MC (2022). Acoustic emission and damage characteristics of granite under graded cyclic loading. Arab. J. Geosci..

[39] Miao SJ, Liu ZJ, Zhao XG, Huang ZJ (2021). Energy dissipation and damage characteristics of Beishan granite under cyclic loading and unloading. Chin. J. Rock Mech. Eng..

[40] Chen X, Lin J, Cao GY, Yang Y, Yin JC, Fan H (2022). Energy evolution and damage characterization of sandstone under cyclic loading and unloading. Chin. Sci. Technol. Eng..

[41] Ye DY, Wang ZL (2001). A new approach to low-cycle fatigue damage based on exhaustion of static toughness and dissipation of cyclic plastic strain energy during fatigue. Int. J. Fatigue.

[42] Liu XS, Ning JG, Tan YL, Gu QH (2016). Damage constitutive model based on energy dissipation for intact rock subjected to cyclic loading. Int. J. Rock Mech. Min. Sci..

[43] Liu Y, Dai F, Dong L, Xu NW, Feng P (2018). Experimental investigation on the fatigue mechanical properties of intermittently jointed rock models under cyclic uniaxial compression with different loading parameters. Rock Mech. Rock Eng..

[44] Jin F, Jiang M, Gao X (2004). Defining damage variable based on energy dissipation. Chin. J. Rock Mech. Eng..

[45] Jia ZQ, Xie HP, Zhang R, Li CB, Wang M, Gao MZ, Zhang ZP, Zhang ZT (2020). Acoustic emission characteristics and damage evolution of coal at different depths under triaxial compression. Rock Mech. Rock Eng..

[46] Ritter A, Muñoz-Carpena R (2013). Performance evaluation of hydrological models: Statistical significance for reducing subjectivity in goodness-of-fit assessments. J. Hydrol..

